# Impact of electro-acupuncture on EAAT2 and NMDAR-2B expression in goats with visceral hypersensitivity

**DOI:** 10.1016/j.heliyon.2024.e40700

**Published:** 2024-12-02

**Authors:** Adnan Hassan Tahir, Yi Ding, Juan Wan, Manoj Kumar Shah, Habibullah Janyaro, Xiao-Jing Li, Ming-Xing Ding

**Affiliations:** a College of Veterinary Medicine, Huazhong Agricultural University, Wuhan, Hubei, China; b Faculty of Veterinary and Animal Sciences, PMAS-Arid Agriculture University, Rawalpindi, Pakistan; c Gannan Innovation and Transformation Medical Research Institute, First Affiliated Hospital, Gannan Medical University, Ganzhou, Jiangxi, China; d Department of Pharmacology and Surgery, Institute of Agriculture and Animal Science, Rampur Chitwan, Nepal; e Shaheed Benazir Bhutto University of Veterinary and Animal Sciences, Sakrand, Pakistan; f School of Medicine, Shanghai University, Shanghai, China

**Keywords:** Electro-acupuncture, Inflammatory bowel disease, Ileitis, TNBS, Visceral hypersensitivity, NMDAR-2B, Glutamate transporter EAAT2, Goat

## Abstract

**Objective:**

This study evaluates the effect of electro-acupuncture (EA) on visceral hypersensitivity (VH) and the expression of N-methyl-D-aspartate receptor-2B (NMDAR-2B) and glutamate transporter EAAT2 in goats.

**Methods:**

Twenty-four goats were divided into four groups: saline, 2, 4, 6-Trinitrobenzenesulfonic acid (TNBS), TNBS + EA, and sham EA. EA was administered at Zusanli (ST36) with 60 Hz and 1–3 mA on specified days. Electromyography (EMG) recorded visceromotor response to colorectal distention (CRD). Spinal cords were collected for immunohistochemistry, western blotting, and RT-PCR. The ileum was examined histologically.

**Results:**

The repeated EA administration significantly attenuated VH (P < 0.05) in TNBS-treated goats without similar effects in the sham group. NMDAR-2B expression increased (P < 0.01), and EAAT2 expression decreased (P < 0.01) in the TNBS group compared to saline. EA increased the EAAT2 and decreased the NMDAR-2B expression (P < 0.01) compared to TNBS, with no change in the sham-EA group.

**Conclusion:**

EA may alleviate VH by upregulating EAAT2 and downregulating NMDAR-2B in the spinal cord of TNBS-treated goats, indicating its potential for treating chronic visceral pain in gastrointestinal disorders.

## 1 Introduction

Functional abdominal pains are caused by major intestinal disorders, such as irritable bowel syndrome (IBS) and inflammatory bowel disease (IBD) in humans and animals. It is established that most patients with these intestinal disorders enhanced the perception of pain or visceral hypersensitivity (VH) in response to distension of the gut lumen [1,2]. VH is considered as a biological marker of these disorders. The exact cause of VH is unknown, but several mechanisms have been postulated that include triggering events such as genetic, psychological, inflammatory, or post-injury sensitization [[3], [4], [5]]. VH not only occurs during acute flares of gastrointestinal inflammation but also exits during its remission [6,7]. The recurrent or persistent VH is one fundamental cause for patients to seek medical help. The drugs targeted for relieving patient VH are analgesics (opiates, nonsteroidal anti-inflammatory drugs), antispasmodics, and antidepressants. Still, their long-term uses have been shown to have side effects such as inadequate pain relief, tolerance, and gastrointestinal and cardiovascular toxicity [8]. Some complementary and alternative therapies (such as acupuncture) have come into use, which have specific curative effects and fewer side effects compared to pharmacotherapy [[9], [10], [11]]. Clinically, electro-acupuncture (EA) has been used to attenuate visceral pain [12,13]. In addition, some experimental studies [[14], [15], [16]] have shown that EA is important in reducing chronic VH. These studies recommend that EA may be useful as a supplementary or alternative therapy for VH in patients with gastrointestinal disorders. It is necessary to find more conclusive information to authenticate EA's effectiveness and understand its underlying mechanism.

There is a strong covenant that VH is associated with spinal cord sensitization, which involves different excitatory neurotransmitters [[17], [18], [19]]. Glutamate has a predominance in VH. Glutamate is usually cleared from the synaptic endings by sodium-dependent and high-affinity glutamate transporters in both glial and neuron cells. The pharmacological inhibition of these transporters in the spinal cord causes increased spinal glutamate neurotransmitters in the synaptic endings [20] and enhanced nociceptive behaviors and hypersensitive response to thermal or mechanical stimuli [20,21]. There is a very important link between glutamate transporters and N-methyl-D-aspartate receptors (NMDARs). The inhibition of glutamate transporters causes an increase in NMDARs subunit 2B (NR2B), which activates and enhances the peripheral inputs [22]. To date, five human glutamate transporters, excitatory amino acid transporters (EAAT1-5), have been classified [23]. The glutamate transporter EAAT2 (in rodents called GLT1) is prominent among the other glutamate transporters in the mammalian CNS, and it has a key role in dismissing synaptic transmission and protecting the neurons from glutamate neurotoxicity [[24], [25], [26]]. The work by Lin et al. showed that EAAT2 is a major mediator in glutamate clearance and attenuates the visceral nociceptive response in mice [27]. He further investigated that upregulation of the GLT1 in the spinal cord in rats reduced the visceral nociceptive response and hyperalgesia [27].

NMDA receptors, glutamate-gated ionotropic channels in the neurons, receive primary afferent inputs and play an energetic role in the initiation and maintenance of the spinal cord sensitization [[28], [29], [30]]. Previous studies have shown that the increase in NMDA receptor expression in the spinal cord was associated with hypersensitivity in different pain assessment tests, such as thermal and mechanical stimuli and colonic distension in TNBS-induced colitis rats [31]. NMDA receptors comprise three subunits: NR1, NR2A-D, and NR3. NR1 is an essential functional subunit, while NR2A, 2B, 2C, and 2D are the modulatory subunits. The role of NR3 remains indistinct [[32], [33], [34]]. Predominantly, The NR2B in the spinal cord is mainly responsible for the hyper-sensitization [35,36]. Studies show that the intrathecal administration of a selective antagonist (R_O_ 25–6981) for NR2B subunit can prevent mechanical allodynia, neuropathic pain, and visceral pain [[37], [38], [39]]. Luo et al. produced a rat model of chronic visceral pain and found that NR2B in the spinal cord was activated via tyrosine phosphorylation [39]. This leads to the assumption that increased NR2B and its phosphorylation are involved in the development of VH.

Different findings provide evidence that attenuation of VH can be caused by repetitive applications of EA. Although EAAT2 and NR2B are involved in desensitization and development of VH, respectively, whether EA relieves VH by modifying EAAT2 or NR2B is not clear. In the present study, we evaluated the effect of EA on Glutamate transporter EAAT2 and NMDA-NR2B receptor and its phosphorylation expression in the spinal cord after TNBS-induced ileitis in goats.

## 2 Materials and methods

### 2.1 Animal and grouping

A comprehensive health assessment was conducted on goats to ensure their suitability for the study. Twenty-four clinically healthy goats, comprising 12 males and 12 females, each approximately one year old, were carefully selected for participation in the trial. The goats were then randomly divided into four equally sized groups, namely the saline group, TNBS group, TNBS + EA group, and sham EA group, having six animals each.

The animals were maintained under uniform nutritional and management conditions throughout the study. They underwent a deworming process and were given one week to acclimatize to their surroundings before the commencement of the experiment.

### 2.2 Induction of ileitis

Before the initiation of the experiment, a fasting period of 12 h was observed in the goats to mitigate the risk of anesthesia-induced regurgitation and respiratory complications. Essential baseline parameters were meticulously recorded, such as respiratory rates, pulse rates, and body temperature. Following this, intramuscular injections of atropine (0.05 mg/kg, sourced from Tianjin Xinzheng Pharmaceutical Group Co., Ltd., China) and etamsylate (15 mg/kg, from Shandong Fangming Pharmaceutical Co., Ltd., China) were administered to the goats, allowing a 15-min interval before the commencement of anesthesia.

To induce anesthesia, a combination of Xylazine (from Hebei Gaocheng Sihai Pharmaceutical Industry Co., Ltd., China) and ketamine (from Yao Pharma Co., Ltd, China) was intramuscularly administered at doses of 0.10 mg/kg and 5 mg/kg, respectively. The trachea was intubated to prevent any aspiration of ruminal contents, and the maintenance of anesthesia was achieved through the intravenous administration of ketamine HCl at a rate of 0.5 mg/kg/min. During the procedure, careful monitoring of anesthesia depth involved assessing eyelid and corneal reflexes and responses to stimuli applied to the coronary hooves. A 6-cm incision was then performed on the right flank abdomen, and the distal part of the ileum was exteriorized.

In the case of the TNBS, TNBS + EA, and Sham groups, a 1.2 mL solution containing 2, 4, 6-Trinitrobenzenesulfonic acid (TNBS; sourced from Sigma Aldrich Company, USA) and consisting of 30 mg of TNBS dissolved in 40 % ethanol was injected into the ileum wall through five points, positioned approximately 15 cm proximal to the ileocecal junction, using a 30-gauge needle. The TNBS dosage used in this study was determined based on previous research, wherein it consistently induced significant inflammation in the affected ileum. This inflammation was characterized by notable mucosal ulceration, increased wall thickness, and elevated levels of neutrophils, mast cells, MPO, cytokines, and VH [13,40]. For the Saline group, an equivalent volume of saline was administered following the same standardized procedure as the other experimental groups. Afterward, the intestine was carefully returned to the abdominal cavity, and the abdominal wall and peritoneum were sutured using catgut sutures, while the skin was closed with silk sutures. During the subsequent 1-2-h recovery period from anesthesia, the goats were individually housed and closely monitored. Throughout the recovery phase, the goats resumed their usual grass intake and water consumption, exhibiting normal behavior by the day after the surgical procedure.

To ensure their well-being and minimize potential discomfort, tramadol (10 mg/kg, intramuscular (IM) from Hubei Qianjiang Pharmaceutical Co, Ltd., China) was administered to the goats over three days. Furthermore, meticulous wound care was diligently provided, involving the daily application of iodophor (from Wuhan Xuehuan Sterilization Goods Co., Ltd, China) and erythromycin ointment (from North China Pharmaceutical Co., Ltd, Hebei, China) until complete healing was achieved. Additionally, the animals' weights were recorded at specified intervals on days 0, 3, 7, 10, 13, 16, 19, and 22 post-surgeries to monitor their overall health and progress.

### 2.3 Electroacupuncture stimulation

**Fig. 1:**
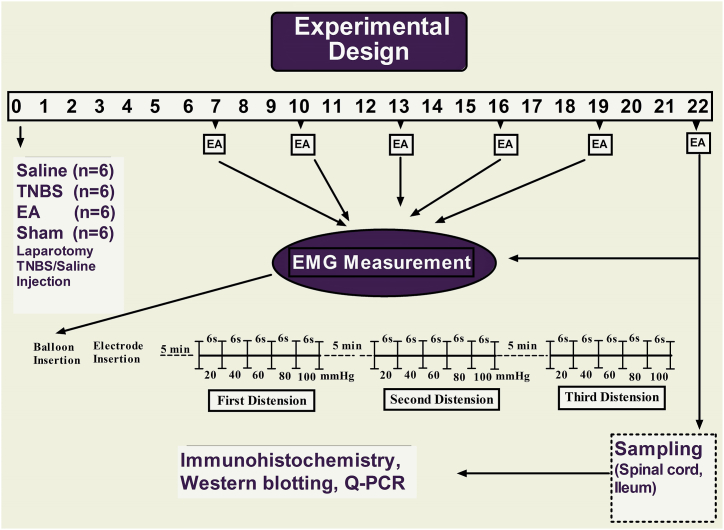
Illustration of the experimental design EMG = Electromyography, EA = Electro-acupuncture.

The animals were slightly restrained on a specially designed table, and their hind legs were exposed bilaterally for EA treatment. EA was applied by a pair of stainless-steel needles (0.45 mm in diameter and 5 cm in length) inserted bilaterally at a depth of approximately 5 mm into two acupoints, zusanli (the upper of the lateral crus, below fibula capitulum, in the groove of toe long extensor and toe lateral extensor muscle) of each hind leg. Each pair of needles was connected with the output terminals of an EA apparatus WQ-6F Electronic Acupunctoscope (Beijing Xindonghua Electronic Instrument Co., Ltd., Beijing, China). Goats in the EA group were undergoing electroacupuncture for 30 min (with frequencies 60 Hz and intensity of 2–3 mA) on days 7, 10, 13, 16, 19, and 22 after TNBS treatment. In the Sham group, needles were inserted into the same acupoints and left for 30 min without receiving electrical stimulations on the same days (Fig. 1). All the experimental goats were immobilized using the same restraint method.

### 2.4 Colorectal distension testing

Colorectal distension testing was conducted to assess visceral hypersensitivity (VH) using electromyography (EMG) in goats at various time points after surgery (7, 10, 13, 16, 19, and 22 days; see Fig. 1). Briefly, before EMG record, two thin needles were inserted 2 cm apart into external oblique muscles at the center of the left abdomen as electrodes, which was attached to an EMG recorder (Nanjing Medease Science and technology Co., Ltd, China) by a wire. EMG amplified and filtered data were processed using MedLab-U/4C501H (Nanjing Medease Science and Technology Co., Ltd, China). A manual distension device was custom-made for the study. The device consisted of a T-connector that connected a polyethylene balloon (12 cm), a vacuum pump, and a sphygmomanometer [15,40]. The lubricated balloon was inserted 10 cm from the anus into the distal colon of the goats. After a 10-min acclimatization period, the balloon was gradually inflated using the pump, and its pressure was measured using the sphygmomanometer. EMG recordings were initiated when the balloon reached 20 mmHg pressure and were continued for 6 s. Subsequently, the pressure was incrementally increased to 40, 60, 80, and 100 mmHg in stages, and EMG was recorded for 6 s at each pressure level. This entire procedure was repeated three times with a 5-min interval between repetitions. The EMG data collected during the testing were analyzed using MedLabV6.3 software (Nanjing Medease Science and Technology Co., Ltd, China) and were expressed in millivolts per second (mv/s).

### 2.5 Sample collections

All animals from each group (6 number) were euthanized on day 22 immediately after the CRD test. The spinal cord at the eleventh thoracic vertebrae (T11) was carefully extracted and divided into two distinct portions. The anterior part was preserved in 10 % buffered formalin for subsequent immunohistochemistry analysis. In contrast, the posterior part was weighed, rapidly frozen in liquid nitrogen, and stored at −80 °C for RT-PCR and western blotting investigations.

The ileum, located approximately 15 cm proximal to the ileocecal junction, was removed and thoroughly flushed with PBS. The ileum segment was then longitudinally dissected and laid flat on a clean drape sheet, allowing for macroscopic examination of mucosal changes. Subsequently, a tissue block measuring 2 × 2 cm was excised and preserved in 10 % buffered formalin for histopathological evaluation. All procedures were conducted with utmost swiftness to minimize any post-mortem alterations.

### 2.6 Macroscopic and microscopic observations

The macroscopic and microscopic lesions were evaluated by two impartial observers blind to the experimental treatments. Macroscopic assessments encompassed factors such as the adhesion of ileal serosa to the intestinal loops, presence of mucosal pseudo-membrane, hyperemia, extent of ulceration, and wall thickness. Histological studies were conducted using routine techniques to process the tissue samples. Microscopic scores were based on the following criteria: crypt depth, extent of inflammatory cell infiltration, submucosal thickness, and the degree of blood vessel congestion. The scoring system ranged from 0 to 10, as described previously [40].

### 2.7 Immunohistochemistry

The expression of EAAT2, NR2B subunit, and NR2B phosphorylation were examined through immunohistochemistry. The goat's spinal cord fragment was taken from 10 % formalin and embedded in a paraffin block, sectioned at 5 μm, mounted on polylysine-coated slides, deparaffined, and rehydrated sequentially. Endogenous peroxide activity was quenched with 3 % H_2_O_2_ for 10 min at room temperature. Washed with distilled water three times, slides were treated with sub-boiling citrate buffer to unmask antigens. Then, the sections were washed with TBST (pH 7.4), blocked with 5 % bovine serum, and incubated at 37 °C for 30 min. Three slides for each substance were incubated with rabbit polyclonal antibody for EAAT2 ***(H-85): sc-15317 (***Santa Cruz Biotechnology, Inc. USA), and six slides for mouse monoclonal antibody for NR2B (1:500, Abcam, USA), or phosphorylated NR2B rabbit anti-NR2B (pTyr1472) polyclonal antibody (1:500, Abcam, USA) at four °C for overnight. Three slides were treated with PBS instead of the primary antibody solution and used as the negative control. Following three rinses, the sections were washed with TBST and incubated with the biotinylated secondary antibodies (mouse anti-rabbit IgG, 1:400 and rabbit anti-mouse IgG, 1:500; Boster Biological Technology Co., Ltd Wuhan, China) at 37 °C for 30 min. Diaminobenzidine (DAB) was applied. All the sections were dehydrated with graded ethanol series and cleared with xylene, cover-slipped, and air-dried. Images were captured at 20 × objective lens using a digital microscope with the camera (Nikon ECLIPSE 80I, Nikon Corporation, and Tokyo, Japan). The positive cells were dyed with a yellow-brown color.

### 2.8 Western blotting

The SCDH tissue was ground in liquid nitrogen, and protein extraction was carried out from the ground tissue using the RIPA buffer according to the manufacturer's guidelines (Beyotime Biotech, Nantong, China). The protein concentration was quantified using a NanoDrop (Thermo Fisher Scientific, Inc., USA). The exact amounts of protein (40 μg) from each sample were loaded into 8 % SDS polyacrylamide gel for NMDA-NR2B and 10 % SDS polyacrylamide gel for EAAT2, respectively and transferred to a PVDF membrane using the Mini-PROTEIN Tetra Cell (Bio-Rad, Hercules, CA, USA). The membrane was then blocked for 1 h at room temperature in 5 % skimmed milk PBST blocking buffer and then treated overnight with corresponding rabbit anti-EAAT2 (1.300, Santa Cruz Biotechnology, Inc. USA), mouse anti-NR2B (1:500, Abcam, USA), or rabbit anti-pNR2B (1:500, Abcam, USA) and rabbit anti-beta-actin (1:300, Santa Cruz Biotechnology, Santa Cruz, CA, USA) at four °C in 1 % milk PBST. After three 10-min washings with PBST, the membrane was further incubated at room temperature for 1 h with horseradish peroxidase (HRP)-conjugated mouse anti-rabbit IgG (1:3000) and rabbit anti-mouse IgG (1:3000) in 1 % milk PBST. The HRP signal was detected by incubation with Enhanced Chemiluminescent Substrate (Beyotime Shanghai, China), and the bands were visualized using the luminescent Image Analyzer (Image Quant, USA). Further analysis of bands was performed with Quantity One software (Bio-Rad) on scanned images of the membrane. Beta-actin was employed as an internal control, and the values of these substances were expressed as the ratio of the optical density of the bands to the density of the corresponding beta-actin band.

### 2.9 RT-PCR

RT-PCR was employed to investigate the impact of electro-acupuncture on gene expression of EAAT2 and NR2B subunit in the SCDH. Total RNA was extracted from the SCDH of each sample by using Trizol reagent (Invitrogen, Carlsbad, CA, USA) and reverse-transcribed into cDNA using a First-Strand cDNA Synthesis Kit (TOYOBO, Osaka, Japan). To ensure sample integrity and reaction fidelity in quantitative PCR (Q-PCR), the housekeeping gene GAPDH expression was considered a control. The polymerase chain reaction was conducted using specific primers based on the following sequences: for GAPDH forward GTCTTCACTACCATGGAGAAGG reverse TCATGGATGACCTTGGCCAG; for EAAT2 forward primer GGTCAGTGCCAACAAC reverse GAGGACGAGTGGGACT; and for NR2B forward CTGCCACAATGAGAAGAACGAGG reverse GCCAGAACAGACACCCATAAAGC. Gene expression quantification was done using the Step-One-Plus™ Real-Time PCR-System (Applied-Biosystems, CA, USA) in connection with the SYBER Green RT-PCR kit (Takara-Dalian, China). Thermal cycling parameters were as follows: 95 °C for 30 s, 40 amplification cycles at 95 °C for 8 s, 59 °C for 30 s, and 72 °C for 30 s. The mRNA of EAAT2 and NR2B relative to GAPDH were determined using the 2^−delta-Ct^ method, where delta C_t_ = C_t_ target gene - C_t_-GAPDH.

### 2.10 Statistical analysis

Experimental data were expressed as mean ± standard deviation (SD). Statistical analyses were performed using SPSS version 18.0 (SPSS Inc., Chicago, IL, USA). Comparison between groups was performed using the one-way analysis of variance (ANOVA) followed by Bonferroni's post hoc test. The differences in macro- and microscopic scores among the groups were analyzed with the Mann-Whitney Test. P < 0.05 was considered statistically significant.

## 3 Results

### 3.1 Macroscopic and microscopic changes of the TNBS-treated ileum

Macro- and microscopic changes were not found on day 22 after ileal saline injections. The ileal mucosa of the TNBS group, EA-group, and sham group exhibited mild congestion and wall thickening with no evident or apparent lesions observed in the adjacent viscera such as intestines and mesentery, etc across all groups. The macroscopic score did not show any difference (*P* > 0.05) among the groups on day 22.

**Fig. 2:**
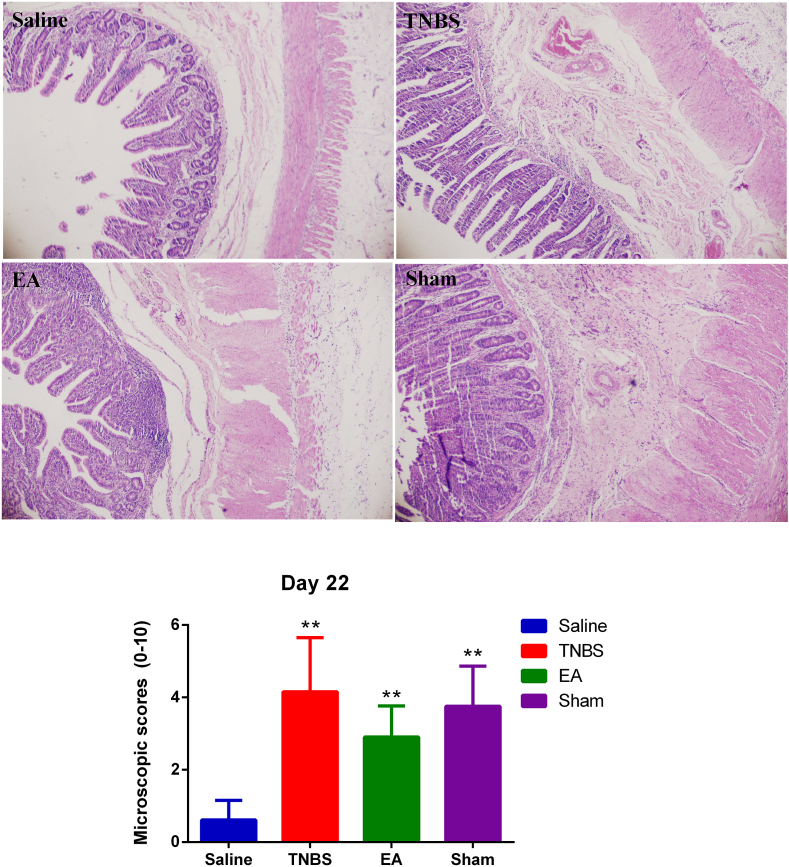
Microscopic lesions and microscopic lesion scores in the ileum (stained with hematoxylin and Eosin) of goats on day 22 after treatments. The representative images of the different groups were taken at 10×. There was no significant inflammation in the Saline group, moderate infiltration of inflammatory cells and ulceration, and wall thickness in the TNBS, EA, and Sham groups. The values are means ± SD, n = 6. ∗∗P < 0.01 TNBS, EA, and sham group vs. Saline group**.**

In all the TNBS-treated groups, the ileal wall exhibited moderate infiltration of inflammatory cells and granuloma in the submucosa and muscular layer on day 22. Microscopic lesion scores in the TNBS, EA, and Sham groups were 4.15 ± 1.50, 2.90 ± 0.86, and 3.75 ± 1.11, respectively, significantly higher (*P* < 0.05) than those observed in the Saline-group (0.61 ± 0.54) (Fig. 2).

### 3.2 Visceromotor response to colorectal distension

**Fig. 3:**
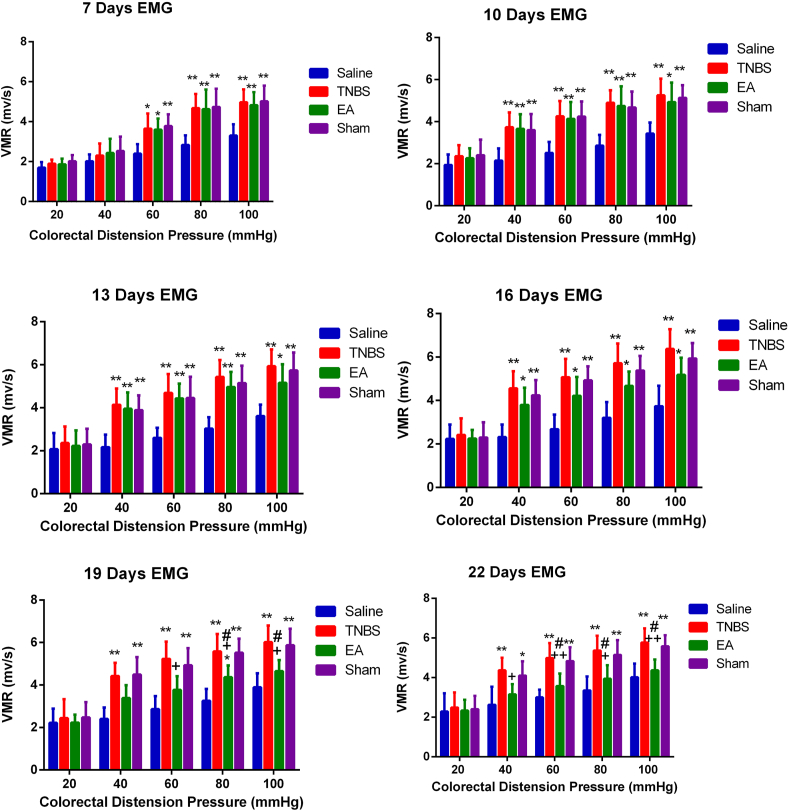
Visceromotor responses (VMR) to goats' colorectal distension (CRD) at days 7, 10, 13, 16, 19, and 22 after surgery. The values are means ± SD, n = 6. ∗P < 0.05, ∗∗P < 0.01 TNBS, EA, and sham group vs. Saline group, ^+^P < 0.05, ^++^P < 0.01; EA, sham group vs. TNBS ^#^P < 0.05, ^##^P < 0.01; TNBS, sham vs. EA.

EMG was utilized to assess the VH. EMG activity was measured to reflect the VMR to graded CRD at various pressure levels (20, 40, 60, 80, and 100 mmHg). During CRD, the goats exhibited behavioral signs such as rapid breathing, guarding, restlessness, tail wagging, posture change, and head movement towards the abdomen. VMRs in the TNBS-treated and Sham goats were pressure-dependently higher (P < 0.05) than during pressures 40–100 mmHg in the control at days 7, 10, 13, 16, 19, and 22. There was no difference (P > 0.05) in EMG between the TNBS and Sham groups throughout the experiment. EA caused EMG values to decrease (P < 0.05) in response to 60, 80, and 100 mmHg on day 16 and to 40, 60, 80, and 100 mmHg on day 19 and day 22. (Fig. 3). These findings suggest that EA attenuated the VMR in goats experiencing TNBS-induced-ileitis VH.

### 3.3 Effect of EA on EAAT2 expression in the spinal dorsal horn

**Fig. 4:**
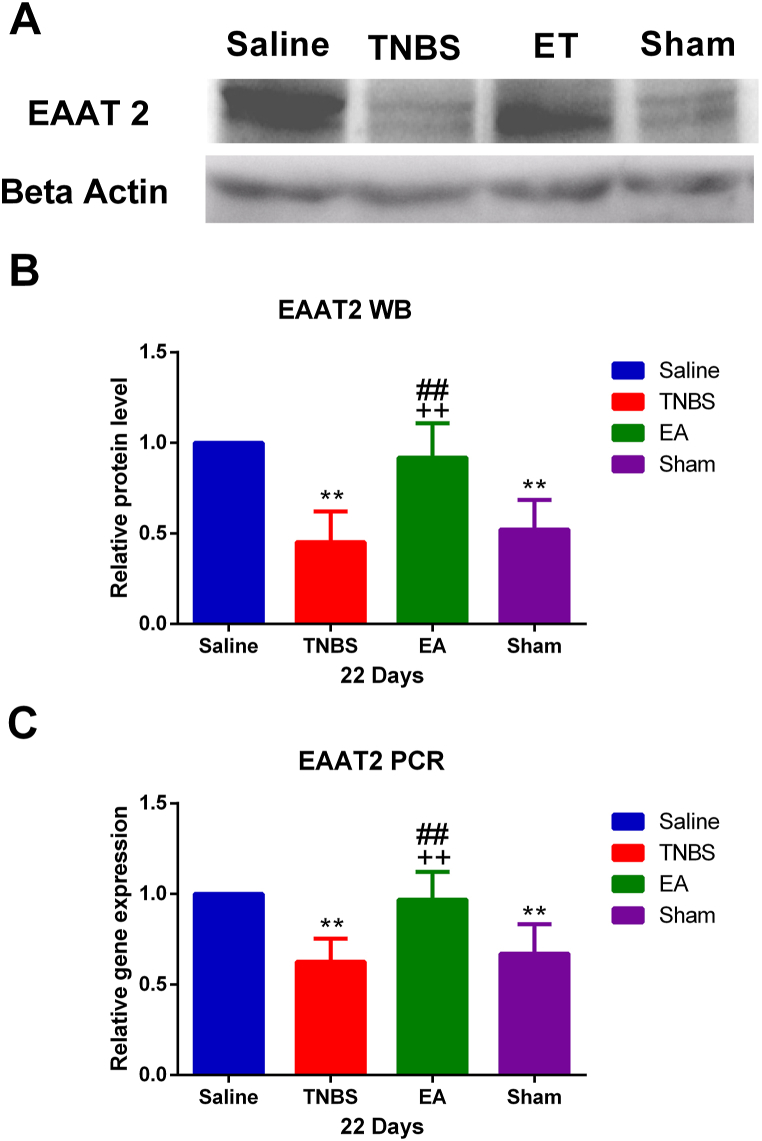
Effects of Electro-acupuncture (EA) on excitatory amino acid transporter 2 (EAAT2) expression in the spinal cord dorsal horn of the goats. Samples were collected from the spinal cord of the T11 segment on day 22 after surgery. (A) Western blotting EAAT2, in response to EA in the spinal cord of the goats. Electrophoresis was performed in the same conditions for all the samples. (B) The expression of EAAT2 was represented by Quantity One software (Bio-Rad) on scanned membrane images. (C) Relative gene expression of EAAT2 using q PCR. The values mean ± SD, n = 6. ∗∗P < 0.01 TNBS, EA, and sham group vs. Saline group, ^++^P < 0.01; EA vs. TNBS ^#^P < 0.05, ^##^P < 0.01; EA vs sham. For uncropped pictures of gels, please see Supplementary Fig. 4.

At day 22 after TNBS administration, EAAT2 gene expression decreased (P < 0.01) compared with the Saline group. EAAT2 gene expression in the EA group was higher (P < 0.01) than in the sham or TNBS group (Fig. 4C). However, there was no change in EAAT2 mRNA between the Sham and TNBS groups. EAAT2 protein changed similarly to its mRNA; TNBS decreased EAAT2 protein while EA increased (P < 0.01) EAAT2 protein at day 22 of the experiment (Fig. 4A and B).

**Fig. 5:**
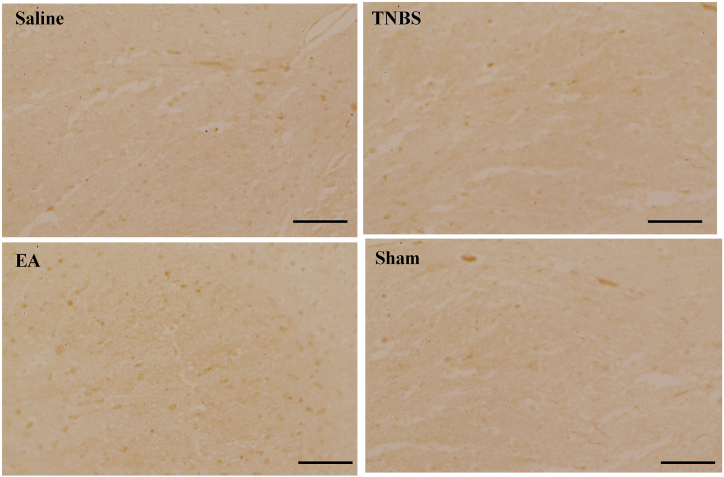
Excitatory amino acid transporter 2 (EAAT2) positive immunoreactivity in the dorsal horn of the spinal cord at day 22 (20 × ) in Saline, TNBS, EA, and Sham groups. Brown stain shows the nuclei of positive cells or cytoplasm. Scale bars = 50 μm.

Immunohistochemistry demonstrated that TNBS or Sham treatment decreased EAAT2 positive cells, whereas EA increased EAAT2 positive cells in the dorsal horn of the spinal cord at day 22 of the experiment (Fig. 5).

### 3.4 Effect of EA on NR2B expression in the spinal dorsal horn

**Fig. 6:**
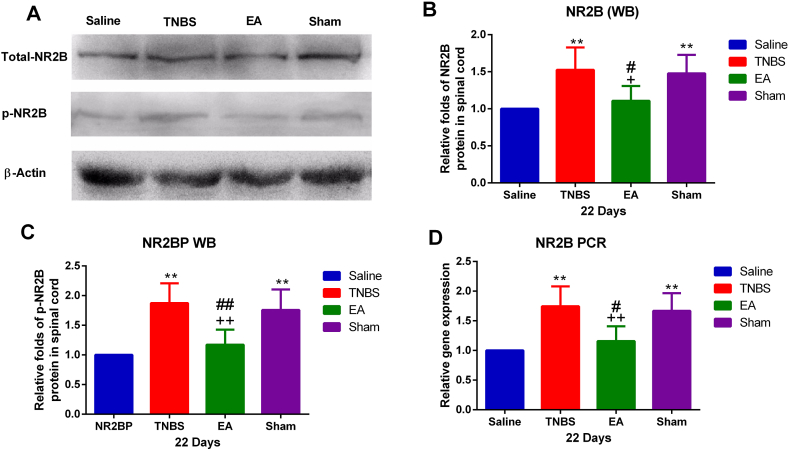
Effect of EA on N-methyl-D-aspartate receptors 2B (NR2B) expression in the spinal cord dorsal horn. Samples were collected from the spinal cord of the T11 segment on day 22. (A) Western blotting of NR2B and p-NR2B in response to EA in the spinal cord of the goats. (B) The representative expression of NR2B was measured using Quantity One software (Bio-Rad) on scanned membrane images. (C) The representative expression of p-NR2B was measured using Quantity One software (Bio-Rad) on scanned membrane images. (D) Relative gene expression of NR2B using q PCR. The values are means ± SD, n = 6. ∗∗P < 0.01 TNBS, EA, and sham group vs. Saline group, ^+^P < 0.05, ^++^P < 0.01; EA vs. TNBS ^#^P < 0.05, ^#^P < 0.05, ^##^P < 0.01; EA vs Sham. For uncropped pictures of gels, please see Supplementary Fig. 6.

NR2B mRNA in the TNBS group increased (P < 0.01) compared with the goats in the Saline group. Repeated application of EA at ST-36 decreased (P < 0.01) NR2B gene expression compared with the TNBS or Sham group. There was no difference between the TNBS group and the sham group (Fig. 6D).

**Fig. 7:**
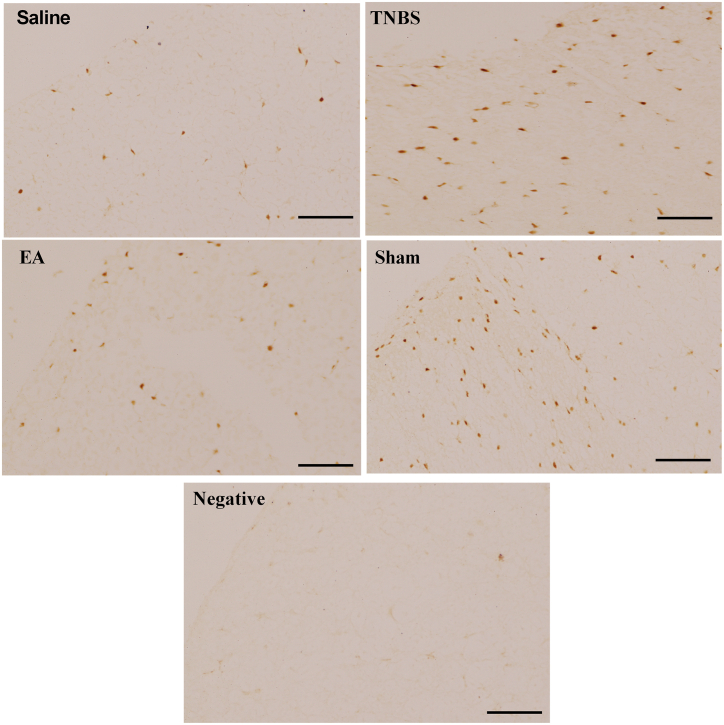
N-methyl-D-aspartate receptors 2B (NR2B) immunoreactivity in the spinal cord dorsal horn at day 22 (20×) in Saline, TNBS, EA, and Sham groups. Brown stain shows the nuclei of positive cells or cytoplasm. Scale bars = 50 μm.

**Fig. 8:**
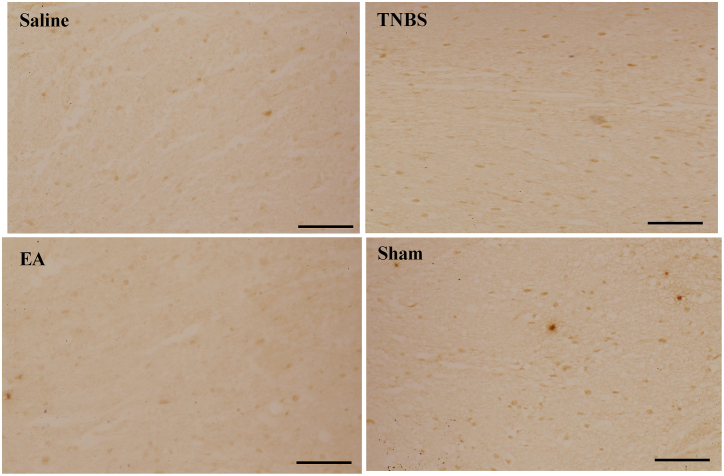
Phosphorylated N-methyl-D-aspartate receptors 2B (p-NR2B) positive immunoreactivity in the spinal cord dorsal horn at day 22 (20×) in Saline, TNBS, EA, and Sham groups. Brown stain shows the nuclei of positive cells or cytoplasm. Scale bars = 50 μm.

Western blotting showed that TBNS-or Sham-treatment enhanced both NR2B and pNR2B levels in the SCDH compared with the TNBS, EA decreased NR2B and pNR2B. There was no difference in NR2B and pNR2B between TNBS-treated and Sham groups (Fig. 6A–C). The distribution of NR2B and pNR2B in the SCDH at day 22 is shown in Fig. 7, Fig. 8. NR2B and pNR2B positive cells changed in the same pattern as their protein expression.

Western blotting analysis showed a significant increase in 180 kDa protein of p-NR2B expression in the TNBS group compared with saline goats. Repeated application of EA at ST-36 decreased (P < 0.01) the p-NR2B expression compared with the TNBS group (Fig. 6A–C). The expression of p-NR2B protein in the TNBS group and Sham group did not significantly change (Fig. 8).

## 4 Discussion

VH is generally regarded as a valid biological indicator for IBS and IBD [9,41]. Colorectal distension is the most extensively used method to study VH in humans and animals [40,42]. There have been subsequent attempts to develop disease models reflecting VH with psychological, mechanical, and chemical stress [13,[43], [44], [45]]. Among them, a chemical model such as TNBS was most frequently used.

Nowadays, TNBS in combination with ethanol (EtOH) has been successfully used in the intra-lumen to induce either ileitis or colitis in mice [46], rats [44], rabbits, guinea pigs [47,48], pigs [49], cats and dogs [50]. In a study, TNBS-induced transient colitis produced somatic and Visceral hypersensitivity in rats up to 28 days [1]. Shah et al. [5], found VH to CRD in TNBS-treated ileum in rats for up to 21 days. Tahir et al. [40] injected TNBS into the ileal wall and found VH for up to 28 days. In the current study, we reproduced this model and found that model goats were more sensitive to innocuous CRD stimulation and exhibited more significant responses to noxious CRD stimulation than normal goats. These results indicate that the visceral perception of TNBS-induced ileitis goats has VH similar to IBS, consistent with previous studies. Therefore, our study supports the conclusion that TNBS ileitis goats lead to VH.

EA has gained widespread popularity worldwide and has demonstrated remarkable effectiveness in treating various diseases and painful conditions in humans and animals [[51], [52], [53]]. The efficacy of Acupuncture-induced analgesia depends on several factors, with the selection of acupoints and frequencies being the most crucial. Studies have shown that EA at ST36 and ST37 attenuates the pain response or hypersensitivity in a somatic pain model [54] and in a colitis model in rats [51]. EA with frequencies between 30 and 100 Hz for cattle has proven effective for analgesia. Previously, Researchers found that EA at 100Hz frequency induced more analgesic effects than 2Hz at ST36 and SP6 acupoints in rats. Cheng et al. [55], and Qiu et al. [52], used different frequencies to stimulate goats and found that 60 Hz induced a higher pain threshold than other frequencies. EA-induced analgesia varies in species. Studies have shown that EA analgesia in goats is superior to that in rats or humans [56]. Therefore, goats should be used as the best animals for studying the underlying mechanism of EA-induced analgesia. In the present study, 60 Hz EA bilaterally used at ST36 acupoints significantly reduced VMR to the colorectal distention, which shows it is effective for attenuating VH.

Central sensitization has a vital role in the initiation and maintenance of VH [57]. However, its underlying mechanism is not fully understood. Different studies demonstrated that NMDA receptors, especially NR2B, mediate the spinal sensitization in the somatic pain model [35,37,58,59]. Lin et al. [60] confirmed with immunohistochemical analysis that NR2B expression was increased in the spinal cord in rats’ IBS model. Furthermore, Lou et al. [39] investigated with western blotting analysis that NR2B antagonist (Ro25-6981) dose-dependently reduced VH in a chronic visceral pain model in rats. In the present study, immunohistochemistry, Western Blot, and Q-PCR analysis confirmed the expression of the NR2B subunit and its phosphorylation in the SCDH. We concluded that TNBS-induced- ileitis increases the spinal cord NR2B subunit and its phosphorylation which is responsible for the central sensitization like in previous Lin and Lou findings in visceral pain. Repeated EA administration decreased the ileitis-induced NR2B expression, phosphorylation, and VMR to CRD in our experiment. These results suggest that EA modifies VH probably through its regulation of NR2B.

Glutamate transporters are important in maintaining the CNS's homeostatic level of extracellular glutamate. Studies have shown that inhibiting glutamate transporters in the spinal cord cause hyperalgesia or increased neural activity. The tricyclic antidepressant amitriptyline upregulates the GLAST and GLT-1 expression in the spinal cord and reduces the pain response [61]. The upregulation of these transporters prevented pathological pain. Roman et al. [62] found that ceftriaxone (activator of GLT-1) attenuated VH and that intrathecal administration of dihydro kainate (a selective GLT-1 antagonist) reversed the antinociceptive response to the bladder distension produced by ceftriaxone. Lin et al. [63] investigated that the up-regulation of GLT-1 attenuated visceral nociception and hyperalgesia via Spinal Mechanisms. These findings support a potential translational approach of GLT-1 upregulation to reduce pain. In our study, EAAT2 gene and protein expression downregulated and enhanced VMR to CRD in TNBS-induced ileitis, which shows that our model is successful for further study. Repeated EA reversed the ileitis-induced downregulation of EAAT2 gene and protein expressions and the enhancement of VMR to CRD, suggesting that EA modified VH probably through regulating EAAT2.

In conclusion, the current study suggests that TNBS injection induced a remarkable VH, increased the expression of NAMDA-NR2B, and decreased the expression of glutamate transporter EAAT2 in the SCDH of goats. Repeated EA treatment can relieve chronic VH, which might be related to the upregulation of EAAT2 and the downregulation of NR2B and pNR2B in the SCDH.

## CRediT authorship contribution statement

**Adnan Hassan Tahir:** Writing – review & editing, Writing – original draft, Methodology, Formal analysis, Data curation, Conceptualization. **Yi Ding:** Writing – review & editing, Methodology, Data curation, Conceptualization. **Juan Wan:** Writing – review & editing, Software, Methodology, Formal analysis. **Manoj Kumar Shah:** Writing – review & editing, Methodology, Formal analysis, Data curation. **Habibullah Janyaro:** Writing – review & editing, Methodology, Data curation. **Xiao-Jing Li:** Writing – review & editing, Methodology, Formal analysis, Data curation. **Ming-Xing Ding:** Writing – review & editing, Supervision, Resources, Project administration, Investigation, Funding acquisition, Conceptualization.

## Ethical statement

Ethical considerations were prioritized, and the study received approval from the Institutional Animal Care and Use Committee of Huazhong Agricultural University, Wuhan, China (HZAUGO-2016-007). Furthermore, the research adhered strictly to the guidelines set forth by the Committee for Research and Ethical Issues of the International Association for the Study of Pain.

## Data availability statement

Data will be made available on request.

## Additional information

No additional information is available for this paper.

## Funding

The study is supported by the 10.13039/501100001809National Natural Science Foundation of China (No. 32172930).

## Declaration of competing interest

The authors declare that they have no known competing financial interests or personal relationships that could have appeared to influence the work reported in this paper.
