# Supplementary material for: Impact of electro-acupuncture on EAAT2 and NMDAR-2B expression in goats with visceral hypersensitivity
Source: Heliyon. 2024 Dec 2;10(23):e40700. doi: 10.1016/j.heliyon.2024.e40700 (PMC11665384; doi:10.1016/j.heliyon.2024.e40700)
Supplement: Multimedia component 1 [file mmc1.docx]

# Supplementary Material

## Supplementary Figure 4:

These are the original images of gels that have been cropped, sliced, or combined to represent them in Figure 4.


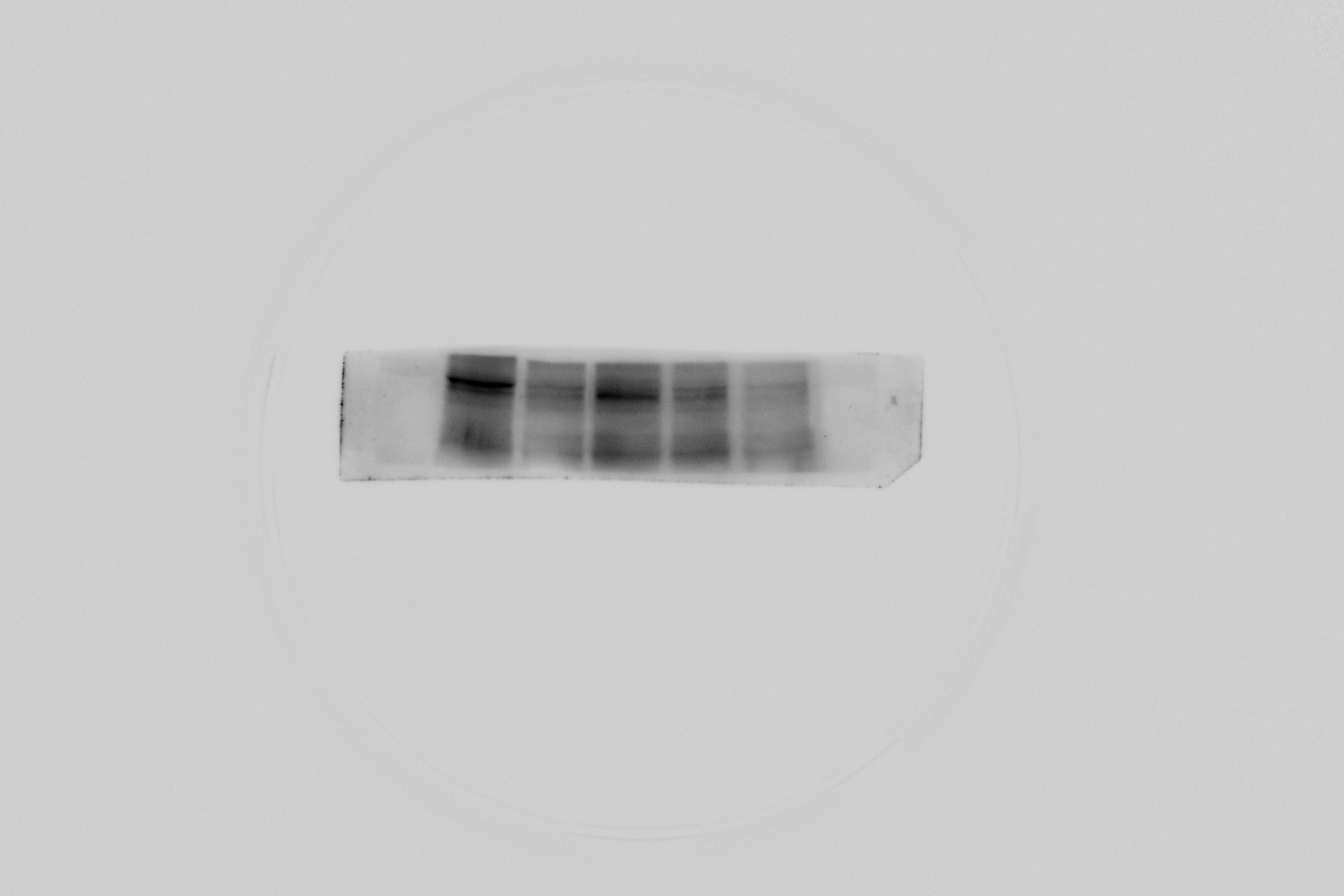


Figure 1: Gel indicating the EAAT2 in Figure 4


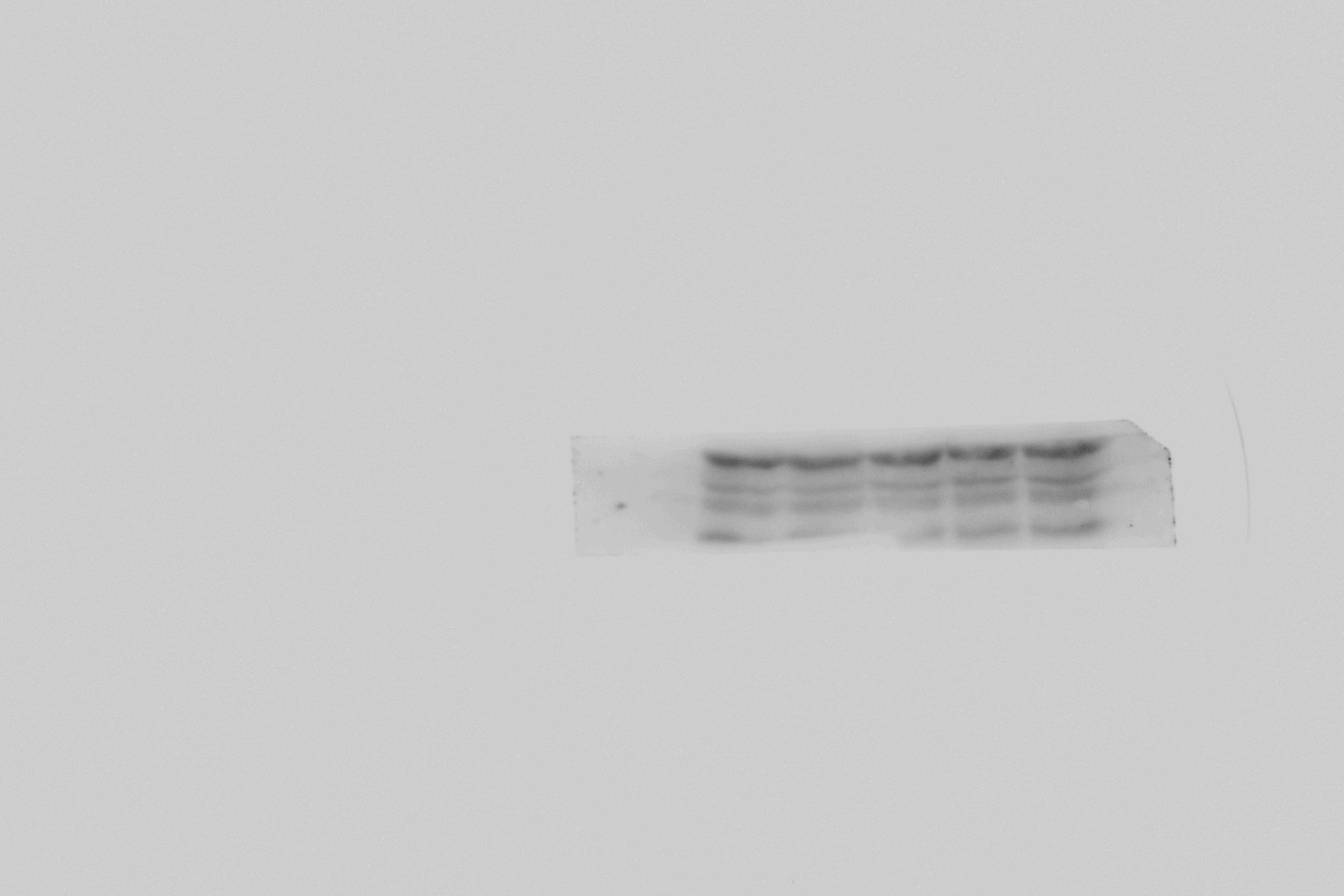


Figure 2: Gel indicating the β-actin in Figure 4

## Supplementary Figure 6:

These are the original images of gels that have been cropped, sliced, or combined to represent them in Figure 4.


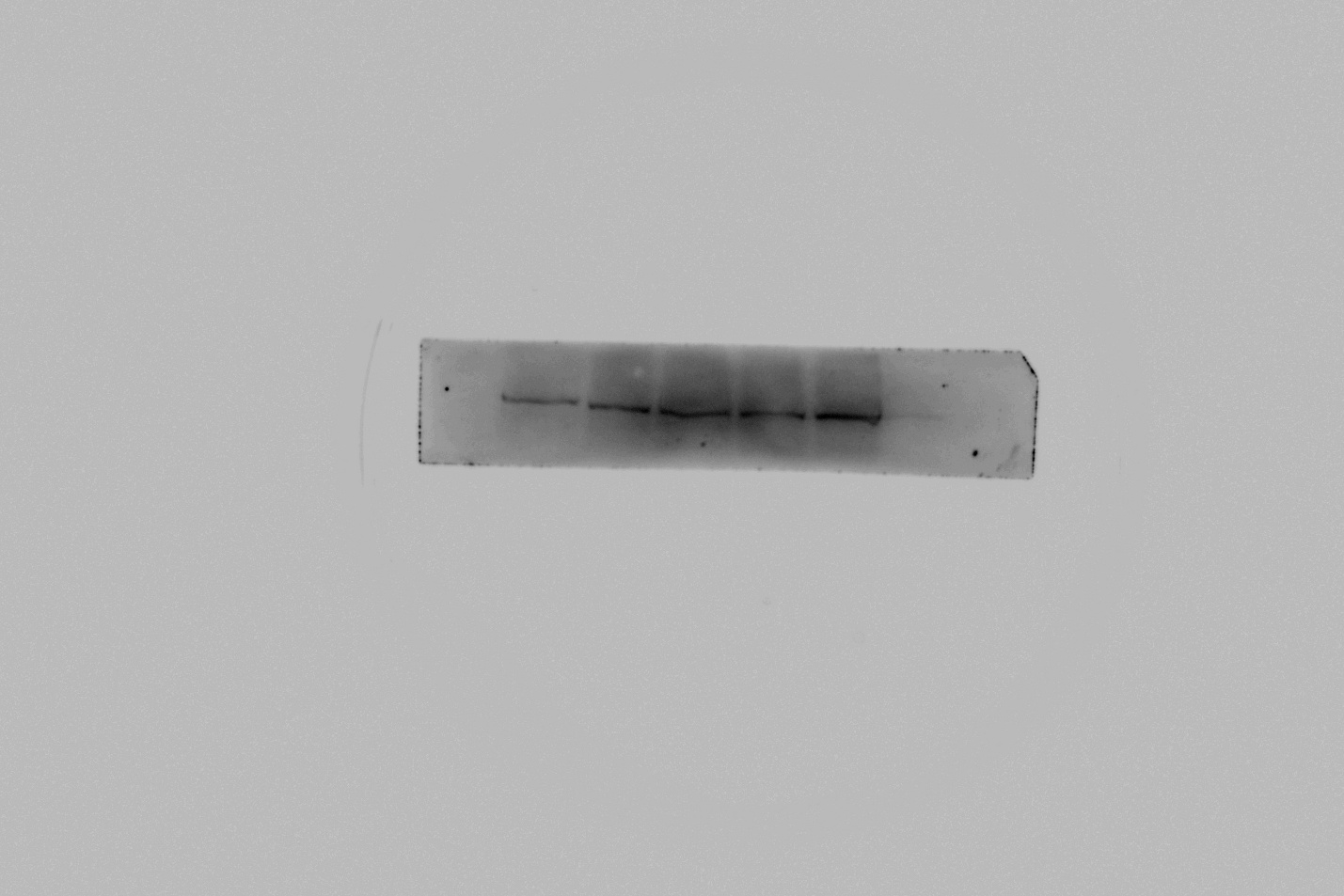


Figure 3: Gel indicating the Total NR2B in Figure 6.


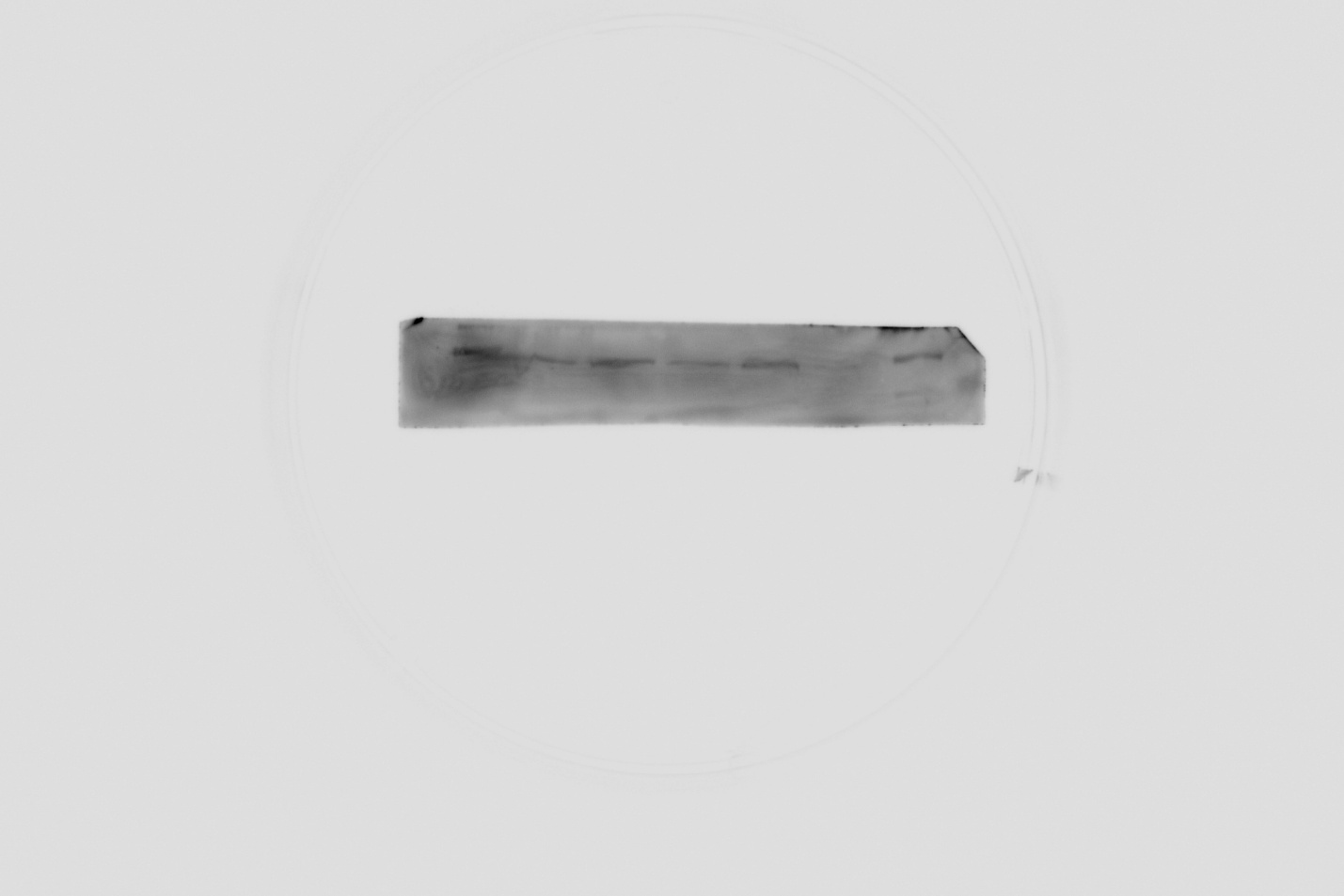


Figure 4: Gel indicating the p-NR2B in Figure 6


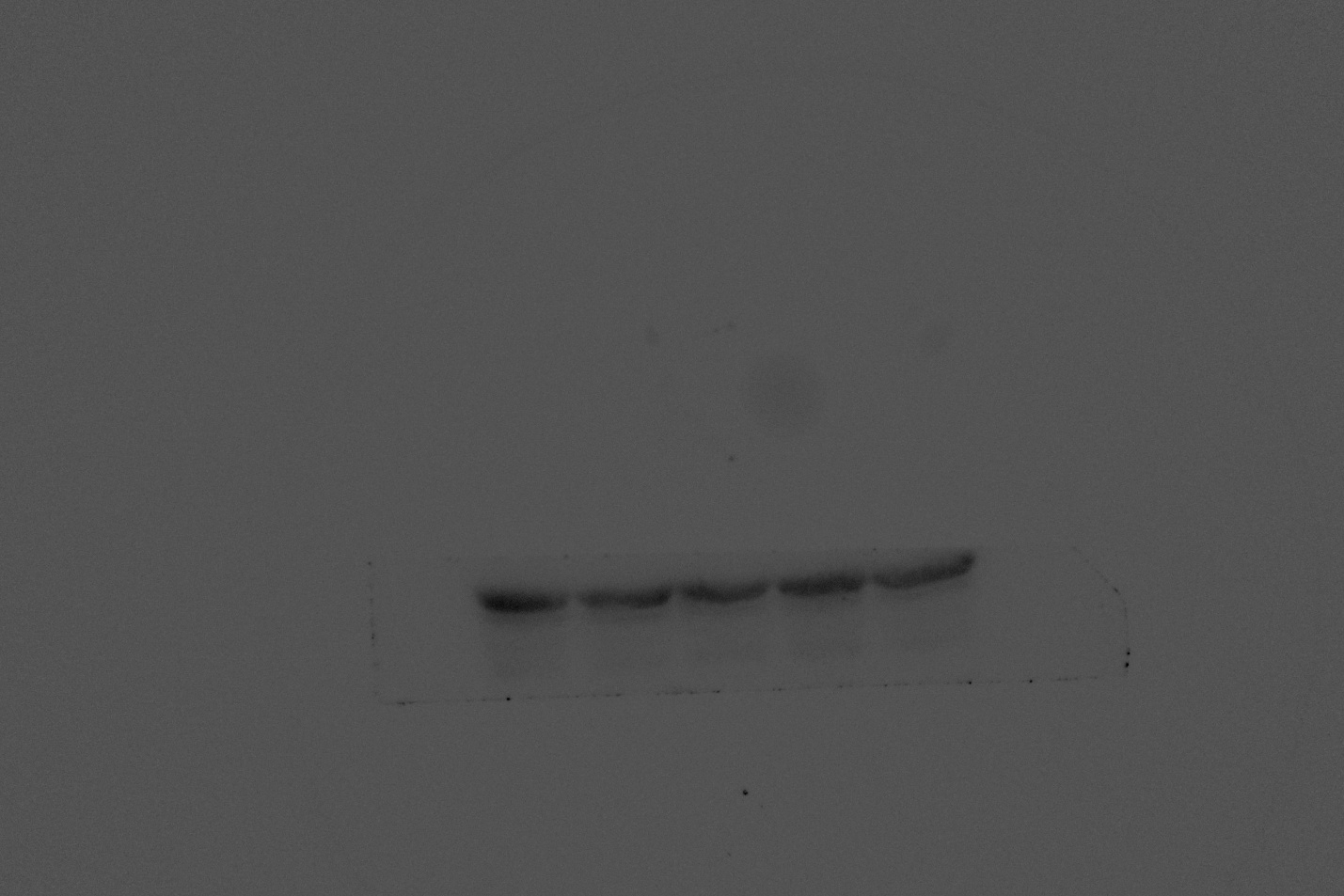


Figure 5: Gel indicating the β-actin in Figure 6.
